# A New Hæmostatic

**Published:** 1885-11

**Authors:** 


### A NEW HÆMOSTATIC.

Dr. Morales, of Barcelona, has found hazeline (an extract of witch-hazel), when locally applied, to act successfully as a styptic in hemophilic (?) patients when all other means, not excepting the thermo-cautery, have failed. Dr. M. is of the opinion that it acts by constricting the vessels as well as by producing coagulation.

Dr. Rothe, of Altenburg, has discovered a reliable hæmostatic in the infusion of *urtica didica* (nettle).—*R. B. D. in Lancet and Clinic.*

